# A Rare Case of Crohn’s Disease Manifesting as a Large Liver Abscess

**DOI:** 10.7759/cureus.3758

**Published:** 2018-12-21

**Authors:** Akriti G Jain, Mohammed FaisalUddin, Idljona Gllava, Dwayne Gordon, Jian Guan

**Affiliations:** 1 Internal Medicine, Florida Hospital, Orlando, USA; 2 Internal Medicine, Deccan College of Medical Sciences, Hyderabad, IND; 3 Internal Medicine, Holmes Regional Medical Center, Melbourne, USA

**Keywords:** crohn's disease, liver abscess, extra-intestinal manifestations of crohn's disease

## Abstract

Liver abscess is a rare complication seen in Crohn's disease (CD) and has been rarely reported. Our aim is to illustrate a case of liver abscess in a patient with CD and the importance of a complete history and physical examination in identifying the potential cause of a hepatic abscess in an immune competent individual in who the diagnosis of CD was apparently ignored for two years before she presented to us. Although the manifestation of a hepatic abscess without any perianal abscess or fistula in CD is extremely rare, recognition of this would facilitate the diagnosis and appropriate management of abscess and CD.

## Introduction

Crohn’s disease (CD) is an idiopathic chronic inflammatory disease of the gastrointestinal tract (GIT), characterised by transmural, non-caseating granulomatous inflammation, affecting most commonly the terminal ileum and/or colon, though any part of the GIT tract may be involved [1]. Pyogenic liver abscess is a very rare hepatobiliary manifestation of CD. Most cases of liver abscess in CD have been seen in young male patients with a long-term and active CD. We aim to present a patient who initially presented with a liver abscess which along with past medical history led to the diagnosis of CD. 

## Case presentation

**Figure 1:**
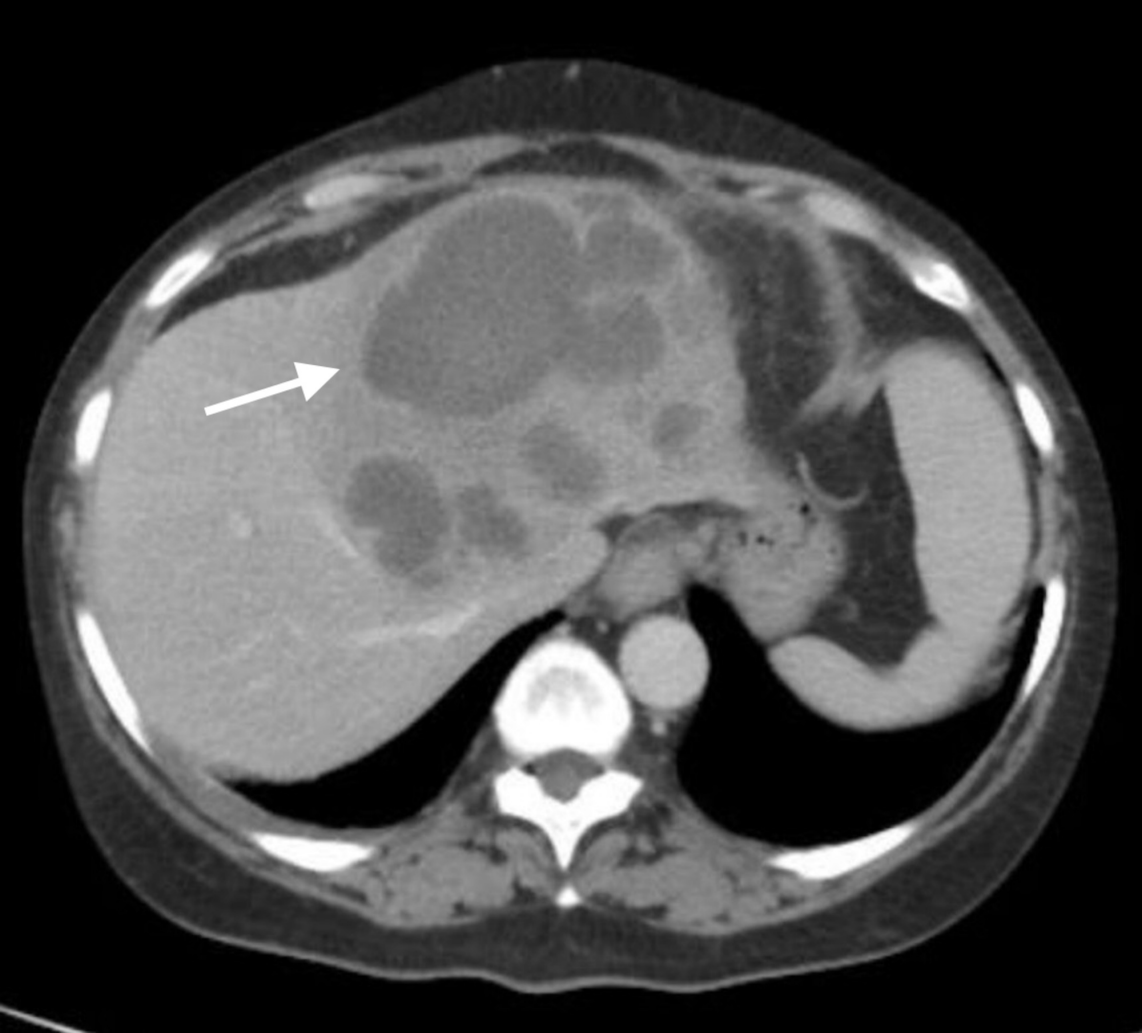
A multi-loculated liver lesion measuring 10 cm x 7.8 cm with multiple hypodense daughter lesions

Here, we report a case of a 55-year-old woman who presented with six months of intermittent high fever, chronic non-bloody watery diarrhea and weight loss. There were no other complaints. She went to another hospital two years ago for chronic diarrhea and abdominal pain, for which she had an incomplete sigmoidoscopy which was normal according to the patient. There was no history of extra-intestinal and hepatobiliary manifestations when she was seen for the first time two years ago. Besides hypertension, she had no significant past medical or surgical history. She denied any travel history, intravenous (IV) drug abuse or a history of chronic intake of immunosuppressants or antibiotics. Her family history was non-contributory. Her vital signs were: temperature 102.2 F, heart rate 105 beats per minute, respiratory rate 18/minute and blood pressure 150/103 mmHg. Physical examination was only significant for mild tenderness in the epigastric/periumbilical area with intact bowel sounds. There was no guarding/rebound tenderness or organomegaly. Laboratory workup revealed elevated white blood cell (WBC) count at 25.91 x 10^9^ cells per liter with a left shift, hemoglobin at 10.1 g/dL and platelets at 462 x 10^9^/L. The basic metabolic panel did not reveal significant electrolytes disturbances. Kidney and liver functions were within normal limits except mild coagulopathy with international normalised ratio (INR) at 1.58 and hypoalbuminemia at 2.9 g/dL. Inflammatory markers erythrocyte sedimentation rate (ESR) and C-reactive protein (CRP) were remarkably elevated. Infectious workup including human immunodeficiency virus (HIV), hepatitis, clostridium difficile, ova/parasites, Entamoeba, Giardia and feces culture with toxin were not suggestive. Stool osmolar gap was indeterminate and celiac workup was negative. Abdominal computed tomography (CT) scan revealed a multiloculated abscess (10 cm x 8 cm) and multiple small abscesses on the left liver lobe (Figure 1).

A clinical diagnosis of the hepatic abscess was made. She was treated with IV fluids and antibiotics including piperacillin-tazobactam and metronidazole. CT-guided biopsy and Jackson-Pratt drainage tube placement were done on day three of hospitalization. Pus-like drainage fluid was drained and culture was positive for Streptococcus viridans. Patient was discharged home with two weeks of IV piperacillin-tazobactam. Follow-up CT abdominal scan at three weeks revealed the resolution of multifocal abscess in the liver. Her vital signs, WBC and CRP at follow-up were within normal limits. The medical report we requested from the other hospital where she had the incomplete sigmoidoscopy finally arrived and the pathology report indicated the diagnosis of CD. She was scheduled for a follow-up colonoscopy for further management of her CD.

## Discussion

CD is a chronic ulcerative disease of the GIT involving the entire thickness of the bowel wall leading to weakness of the effected segmental wall presenting with many extra-intestinal manifestations including uveitis, migratory polyarthritis, sacroiliitis, ankylosing spondylitis, erythema nodosum, and clubbing of the fingertips [2]. Hepatobiliary complications such as pericholangitis, cholelithiasis, steatosis, primary biliary cirrhosis and primary sclerosing cholangitis are rare in CD but commonly occur in ulcerative colitis [3]. Pyogenic liver abscess is an uncommon complication accounting for 3-5 per 100,000 hospital admissions, but it is an important entity due to the potential lethality and complications [4]. Symptoms such as chills, vomiting, fever, right upper abdominal pain, dark-colored urine, clay-colored stool diarrhea mostly occur. On physical examination, right upper quadrant pain is seen in most of the patients, hepatomegaly and icterus can also be found. Splenomegaly may be seen in chronic patients, pleural effusion, empyema, atelectasis and pneumonia may rarely accompany. Liver abscesses when found in patients with CD are usually multiple and occur in young patients with severe or long-term disease [5]. Liver abscess is sometimes overlooked and regarded as reactivation of CD.

There are multiple possibilities which can lead to liver abscess including the formation of fistulas in long standing cases between loops of the intestine or from skin to gut which provides a nidus for the infection to spread to many abdominal organs including the liver parenchyma [6]. Some patients can also develop vascular seeding, either portal or arterial, predominantly from the GIT leading to the carriage of pyogenic organisms to the liver [7]. Patients with CD may also develop liver abscess due to local spread of infection from the abdominal organs or due to ascending infection in the biliary tract. Moreover, patients on long-term antibiotics, malnutrition, hospitalization and steroids or other immunomodulatory medications including infliximab have compromised gut flora which paves a path for other pyogenic bacteria like streptococcus and staphylococcus to grow, which can be the source of infection to spread to the liver through the biliary tract or fistulas tract [8].

Pyogenic liver abscesses most of the time are polymicrobial. Most frequently isolated organisms include *Escherichia coli*, *Klebsiella pneumoniae*, *Streptococcus viridans*, *Staphylococcus aureus*, *Enterococcus faecalis*, Bacteroides spp., Fusobacterium spp., anaerobic streptococci, Actinomyces spp, and rarely Clostridia spp [9]. Early diagnosis is very important to minimize the complications and surgical interventions. Often times CD is missed due to the variability and subtle presentation and symptoms when compared to ulcerative colitis. Mainstay for the diagnosis of liver abscess in patients with CD is taking a thorough clinical history, physical examination and to choose appropriate abdominal imaging modalities such as CT or ultrasound. CT and ultrasound localize abscess in the liver; however, CT scan is superior to ultrasound in determining the location and number of abscesses.

Broad spectrum antibiotics remain the treatment of choice for liver abscess and should be changed based on specific organism isolated from the liver abscess aspirate. An abscess larger than 2 cm should be drained through image guided (CT scan and ultrasound) percutaneous technique.

## Conclusions

In conclusion, CD should be diagnosed promptly and liver abscess should be kept as a differential when the patient presents with symptoms of right upper quadrant pain, fever, nausea, vomiting and weight loss because involvement of the liver significantly increases the rate of mortality.
